# General Principles of Preclinical Study Design

**DOI:** 10.1007/164_2019_277

**Published:** 2020-01-01

**Authors:** Wenlong Huang, Nathalie Percie du Sert, Jan Vollert, Andrew S. C. Rice

**Affiliations:** Institute of Medical Sciences, School of Medicine, Medical Sciences and Nutrition, University of Aberdeen, Aberdeen, UK; NC3Rs, London, UK; Pain Research Group, Faculty of Medicine, Department of Surgery and Cancer, Imperial College London, London, UK

**Keywords:** Experimental bias, Hypothesis generating, Hypothesis testing, In vivo studies, Preclinical research

## Abstract

Preclinical studies using animals to study the potential of a therapeutic drug or strategy are important steps before translation to clinical trials. However, evidence has shown that poor quality in the design and conduct of these studies has not only impeded clinical translation but also led to significant waste of valuable research resources. It is clear that experimental biases are related to the poor quality seen with preclinical studies. In this chapter, we will focus on hypothesis testing type of preclinical studies and explain general concepts and principles in relation to the design of in vivo experiments, provide definitions of experimental biases and how to avoid them, and discuss major sources contributing to experimental biases and how to mitigate these sources. We will also explore the differences between confirmatory and exploratory studies, and discuss available guidelines on preclinical studies and how to use them. This chapter, together with relevant information in other chapters in the handbook, provides a powerful tool to enhance scientific rigour for preclinical studies without restricting creativity.

This chapter will give an overview of some generic concepts pertinent to the design of preclinical research. The emphasis is on the requirements of in vivo experiments which use experimental animals to discover and validate new clinical therapeutic approaches. However, these general principles are, by and large, generically relevant to all areas of preclinical research. The overarching requirement should be that preclinical research should only be conducted to answer an important question for which a robust scrutiny of the available evidence demonstrates that the answer is not already known. Furthermore, such experiments must be designed, conducted, analysed and reported to the highest levels of rigour and transparency. Assessments of research outputs should focus more on these factors and less on any apparent “novelty”.

## 1 An Overview

**Fig. 1:**
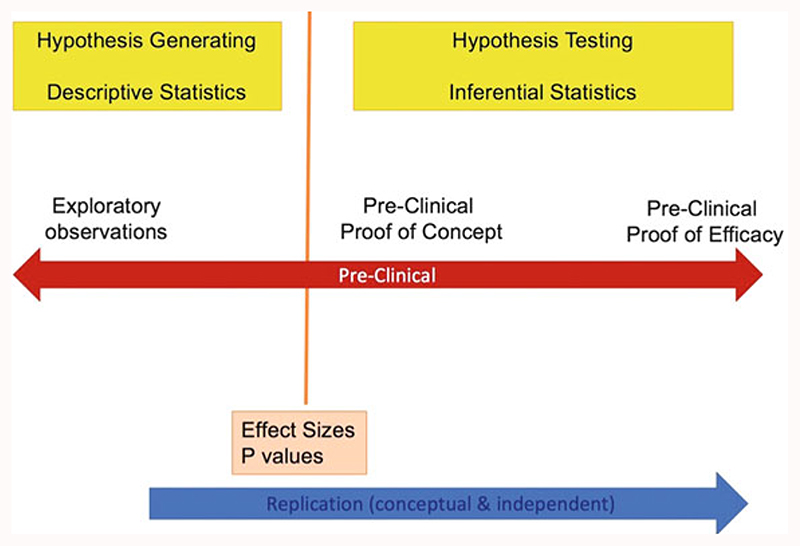
Comparison of exploratory (hypothesis generating) and confirmatory (hypothesis testing) preclinical studies. Descriptive statistics describes data and provides descriptions of the population, using numerical calculations, graphs, and tables. In contrast, inferential statistics predicts and infers about a population using a sample of data from the population, therefore one can take data from samples and make generalisation about a population

Broadly, preclinical research can be classified into two distinct categories depending on the aim and purpose of the experiment, namely, “hypothesis generating” (exploratory) and “hypothesis testing” (confirmatory) research (Fig. 1). Hypothesis generating studies are often scientifically-informed, curiosity and intuition-driven explorations which may generate testable theories regarding the pathophysiology of disease and potential drug targets. The freedom of researchers to explore such innovative ideas is the lifeblood of preclinical science and should not be stifled by excessive constraints in terms of experimental design and conduct. Nevertheless, in order to subsequently assess the veracity of hypotheses generated in this way, and certainly to justify clinical development of a therapeutic target, hypothesis testing studies which seek to show reproducible intervention effects in relevant animal models must be designed, conducted, analysed and reported to the highest possible levels of rigour and transparency. This will also contribute to reducing research “waste” (Ioannidis et al. 2014; Macleod et al. 2014). Chapter “Resolving the Tension Between Exploration and Confirmation in Preclinical Biomedical Research” of the handbook will deal with exploratory and confirmatory studies in details. This chapter will only focus on general design principles for hypothesis testing studies. We will address the issue of design principles for hypothesis-generating studies at the end of this chapter. We advise that when researchers design and conduct hypothesis testing in vivo studies, they should conform to the general principles for the major domains that are outlined in Sect. 4 of the chapter and incorporate these principles into a protocol that can be registered and published. The purpose of using these principles is to enhance scientific rigour without restricting creativity. It is advisable that sometimes there can be exploratory elements within the same hypothesis testing studies; therefore, extra care in terms of applying these principles to reduce experimental biases would be needed before the start of the studies. This chapter will not cover reporting, which will be detailed in chapters “Minimum Information and Quality Standards for Conducting, Reporting, and Organizing In Vitro Research”, “Minimum Information in In Vivo Research”, and “Quality Governance in Biomedical Research” of the handbook.

We would recommend that researchers who conduct hypothesis testing in vivo studies should prepare clear protocols, which include a statistical analysis plan, detailing how they are going to set up measures to address the major domains of experimental biases before the experiments start. Ideally, these protocols should be preregistered and/or published, so that the methods which will be used to reduce the impact of bias are documented in an a priori fashion. The process of peer review of a protocol prior to initiating experiments of course is a valuable opportunity for refinement and improvement. Registering protocols encourages rigour and transparency, even if the protocol is not peer-reviewed. Some journals are open to submissions of these types of protocols, such as BMJ Open Science, and many journals offer the Registered Reports format. In addition, there are online resources that allow researchers to preregister their experimental protocols, such as preclinical. eu and osf.io/registries.

## 2 General Scientific Methods for Designing In Vivo Experiments

Designing an in vivo experiment involves taking a number of decisions on different aspects of the experimental plan. Typically, a comparative experiment can be broken into several component parts.

### 2.1 Hypotheses and Effect Size

The objective is usually to test a hypothesis. On some occasions, two hypotheses may be postulated: the null hypothesis and the alternative hypothesis. The alternative hypothesis refers to the presumption that the experimental manipulation has an effect on the response measured; the null hypothesis is the hypothesis of no change, or no effect. In a statistical test, the p-value reports the probability of observing an effect as large or larger than the one being observed if the null hypothesis was true; the smaller the *p*-value, the least likely it is that the null hypothesis is true. The null hypothesis cannot be accepted or proven true. This also defines the effect of interest, i.e. the outcome that will be measured to test the hypothesis. The minimum effect size is the smallest effect the researcher designs the experiment to be able to detect and should be declared in the protocol; it is set up as the minimum difference which would be of biological relevance. The effect size is then used in the sample size calculation to ensure that the experiment is powered to detect only meaningful effects and does not generate statistically significant results that are not biologically relevant. In many cases, it will be hard to determine the minimum difference of biological relevance as for early stage experiments it might be completely unknown, or translatability between clinical relevance and experimental detection thresholds will be complex. There is no simple and easy answer to this question, but in general, a minimum effect size should be set so one can assume to have a beneficial effect for individuals rather than large cohorts, the difference must be experimentally testable and reasonable to achieve, and should have a rationale for translation into patients in the long run.

### 2.2 Groups, Experimental Unit and Sample Size

In comparative experiments, animals are split into groups, and each group is subjected to different interventions, such as a drug or vehicle injection, or a surgical procedure. The sample size is the number of experimental units per group; identifying the experimental unit underpins the reliability of the experiment, but it is often incorrectly identified (Lazic et al. 2018). The experimental unit is the entity subjected to an intervention independently of all other units; it must be possible to assign any two experimental units to different comparison groups. For example, if the treatment is applied to individual mice by injection, the experimental unit may be the animal, in which case the number of experimental units per group and the number of animals per group is the same. However, if there is any contamination between mice within a cage, the treatment given to one mouse might influence other mice in that cage, and it would be more appropriate to subject all mice in one cage to the same treatment and treat the cage as the experimental unit. In another example, if the treatment is added to the water in a fish tank, two fish in the same tank cannot receive different treatments; thus the experimental unit is the tank, and the sample size is the number of tanks per group. Once identified, experimental units are allocated to the different comparison groups of the desired sample size; this is done using an appropriate method of randomisation to prevent selection bias (see Sect. 3). Each comparison group will be subjected to different interventions, at least one of which will be a control. The purpose of the control group is to allow the researcher to investigate the effect of a treatment and distinguish it from other confounding experimental effects. It is therefore crucial that any control group is treated exactly in the same way as the other comparison groups. Types of control group to consider include negative control, vehicle control, positive control, sham control, comparative control and naïve control (Bate and Clark 2014).

### 2.3 Measurements and Outcome Measures

Measurements are taken to assess the results; these are recorded as outcome measures (also known as dependent variable). A number of outcome measures can be recorded in a single experiment, for example, if burrowing behaviour is measured, the outcome measure might be the weight of gravel displaced, or if neuronal density is measured from histological brain slides, the outcome measure might be the neuron count. The primary outcome measure should be identified in the planning stage of the experiment and stated in the protocol; it is the outcome of greatest importance, which will answer the main experimental question. The number of animals in the experiment is determined by the power needed to detect a difference in the primary outcome measure. A hypothesis testing experiment may also include additional outcome measures, i.e. secondary outcome measures, which can be used to generate hypotheses for follow-up experiments. Secondary outcome measures cannot be used to draw conclusions about the experiment if the experiment was not powered to detect a minimum difference for these outcome measures.

For the purpose of the statistical analysis, outcome measures fall into two broad categories: continuous or categorical. Continuous measures are sometimes referred to as quantitative data and are measured on a numerical scale. Continuous measures include truly continuous data but also discrete data. Examples of true continuous data include bodyweight, body temperature, blood/CSF concentration or time to event, while examples of discrete data include litter size, number of correct response or clinical score. Categorical responses are measured on a nonnumerical scale; they can be ordinal (e.g. severity score, mild/moderate/severe), nominal (e.g. behavioural response, left/middle/right arm maze) or binary (e.g. disease state, present/absent). Continuous responses may take longer to measure, but they contain more information. If possible, it is preferable to measure a continuous rather than categorical response because continuous data can be analysed using the parametric analyses, which have higher power; this reduces the sample size needed (Bate and Clark 2014).

### 2.4 Independent Variables and Analysis

There are many ways to analyse data from in vivo experiments; the first step in devising the analysis plan is to identify the independent variables. There can be two broad types: independent variables of interest which the researcher specifically manipulates to test the hypothesis, for example, a drug with different doses, and nuisance variables, which are other sources of variability that may impact on the outcome measure, but are not of direct interest to the researcher. Examples of nuisance variables could be the day of the experiment, if animals used on different days, or baseline body weight or locomotor activity. Every experiment has nuisance variables. Identifying them at the protocol stage and accounting for them in the design and the analysis, for example, as blocking factors, or co-variables, increase the sensitivity of the experiment to detect changes induced by the independent variable(s) of interest. The analysis plan should be established before the experiment starts and any data is collected; it should also be included in the protocol. Additional analyses can be performed on the data, but if an analysis was not planned before the data was collected, it should be clearly reported as a post hoc or exploratory analysis. Exploratory analyses are at greater risk of yielding false positive results.

## 3 Experimental Biases: Definitions and Methods to Reduce Them

For any researcher who intends to carry out preclinical in vivo studies, it is important to understand what experimental biases are. First, we need to know the definition of bias. It is the inadequacies in the design, conduct, analysis or reporting of an experiment that cause systematic distortion of the estimated intervention effect away from the “truth” (Altman et al. 2001; van der Worp et al. 2010), and it will significantly confound in vivo studies and reduce their internal validity. Sources of bias are multiple and in many cases context dependant. In this overview chapter, it is not possible to give an exhaustive list of potential sources of bias, and it behoves the researcher to systematically identify all potential significant sources of bias for the particular experiment being in planned and to design appropriate mitigation tactics into the protocol. Major known types of biases include selection bias, performance bias, detection bias, and attrition bias. Table 1 gives the definition of each type of bias and describe the methods to reduce them.

Researchers who conduct hypothesis testing in vivo animal work should understand the importance of limiting the impact of experimental biases in the design, conduct, analysis and reporting of in vivo experiments. Experimental biases can cause significant weakness in the design, conduct and analysis of in vivo animal studies, which can produce misleading results and waste valuable resources. In biomedical research, many effects of interventions are fairly small, and small effects therefore are difficult to distinguish from experimental biases (Ioannidis et al. 2014). Evidence (1960–2012 from PubMed) shows that adequate steps to reduce biases, e.g. blinded assessment of outcome and randomisation, have not been taken in more than 20% and 50% of biomedical studies, respectively, leading to inflated estimates of effectiveness, e.g. in the fields of preclinical stroke, multiple sclerosis, Parkinson’s disease, bone cancer pain and myocardial infarction research (Currie et al. 2013; Macleod et al. 2008; Rooke et al. 2011; Sena et al. 2007; van Hout et al. 2016; Vesterinen et al. 2010) and consequently significant research waste (Ioannidis et al. 2014; Macleod et al. 2014, 2015). Therefore, it is imperative that biomedical researchers should spend efforts on improvements in the quality of their studies using the methods described in this chapter to reduce experimental biases which will lead to increased effect-to-bias ratio.

However, it is worth pointing out that the notion that experimental biases could significantly impact on in vivo animal studies is often assumed because they are believed to be important in clinical research. Therefore, such an assumption may be flawed, as the body of evidence showing the importance of bias-reducing methods such as randomisation, blinding, etc. for animal studies is still limited and most of the evidence is indirect. Furthermore, there may also be sources of bias which impact on preclinical studies which are currently unknown. Thus, systematic review and metaanalysis of in vivo studies have shown that papers that do not report bias-reducing methods report larger effect sizes (Vesterinen et al. 2010). However, these studies are based on reported data alone, and therefore there might be a difference between what researchers do and what they report in their publications (Reichlin et al. 2016). Reporting of the precise details of bias reduction methods is often scanty, and therefore accurate assessment of the precise method and rigour of such procedures is challenging. Moreover, those papers that do not report one bias-reducing method, e.g. randomisation, also tend to not report other bias-reducing methods, e.g. blinding and sample size calculation, suggesting that there could be interactions between these methods.

## 4 Experimental Biases: Major Domains and General Principles

In this section, we will describe the major domains, in other words, sources that could contribute to experimental bias if not carefully considered and if mitigating tactics are not included in the design of hypothesis testing experiments before data collection starts. These include sample size estimation, randomisation, allocation concealment, blinding, primary and secondary outcome measures and inclusion/exclusion criteria. General descriptions for these domains (Macleod et al. 2009; Rice et al. 2008; Rice 2010; van der Worp et al. 2010) are shown in the following Table 2. It is important to note that these domains are key things to be included in a protocol as mentioned in Sect. 1.

General principles to reduce experimental bias in each of the above-mentioned domains (Andrews et al. 2016; Knopp et al. 2015) are outlined in the following Table 3.

## 5 Existing Guidelines and How to Use Them

There are resources to assist investigators in designing rigorous protocols and identify sources of bias. Cross-referencing to experimental reporting guidelines and checklists (e.g. ARRIVE (NC3Rs 2018a), the NIH guidelines (NIH 2018a) and the Nature reporting of animal studies checklist (Nature 2013)) can be informative and helpful when planning an experimental protocol. However, it is important to bear in mind that these are primarily designed for reporting purposes and are not specifically designed for use in assisting with experimental design. There are more comprehensive planning guidelines specifically aiming at early experimental design stage. Henderson et al. identified 26 guidelines for in vivo experiments in animals in 2012 (Henderson et al. 2013) (and a few more have been published since, like PREPARE (Smith et al. 2018), developed by the NORECEPA (Norway’s National Consensus Platform for the advancement of the 3Rs), and PPRECISE for the field of pain research (Andrews et al. 2016)). Most of them have been developed for a specific research field but carry ideas and principles that can be transferred to all forms of in vivo experiments. Notable are, for example, the very detailed Lambeth Conventions (Curtis et al. 2013) (developed for cardiac arrhythmia research), from Alzheimer’s research recommendations by Shineman et al. (2011) and generally applicable call by Landis et al. (2012).

The authors of many of these guidelines state that their list might need adaption to the specific experiment. This is pointing out the general shortcoming that a fixed-item list can hardly foresee and account for any possible experimental situation and a blind ticking of boxes ticking of boxes is unlikely to improve experimental design. Such guidelines rather serve an educational purpose of making researchers aware of possible pitfalls and biases before the experimental conduct.

Two examples for a more adaptive and reactive way to serve a similar purpose should be stated: the NIH pages on rigour and reproducibility (NIH 2018b) provide in-depth information and collect important publications and workshop updates on these topics and have a funding scheme specifically for rigour and reproducibility. Second, using the Experimental Design Assistant (EDA) (NC3Rs 2018b; Percie du Sert et al. 2017) developed by the UK’s National Centre for the 3Rs (NC3Rs), a free to use online platform guiding researchers through experimental planning will give researchers the opportunity to adopt guideline and rigour principles precisely to their needs. The researcher creates a flow diagram of their experimental set-up grouped in three domains: the experiment (general questions on hypotheses and aims, animals used, animal strains, etc.), the practical steps (experimental conduct, assessment, etc.) and the analysis stage (e.g. outcome measures, statistical methods, data processing). Unlike a fixed checklist, the EDA checks the specific design as presented by the experimenter within the tool using logic algorithms. The user is then faced with the flaws the EDA identified and can adjust their design accordingly. This process can go through multiple rounds, by that forming a dynamic feedback loop educating the researcher and providing more nuanced assistance than a static checklist can.

While this process, however valid, might take time, the following steps of the EDA actively guide researchers through crucial and complex questions of the experiment, by suggesting fitting methods of statistical analyses of the experiment and subsequently carrying out sample size calculations. The EDA can then also generate a randomization sequence or compile a report of the planned experiment that can, e.g. be part of a preregistration of the experimental protocol.

## 6 Exploratory and Confirmatory Research

It is necessary to understand that there are in general two types of preclinical research, namely, exploratory and confirmatory research, respectively. Figure 1 shows that exploratory studies mainly aim to produce theories regarding the pathophysiology of disease (hypothesis generating), while confirmatory studies seek to reproduce exploratory findings as clearly defined intervention effects in relevant animal models (hypothesis testing). The next chapter will deal with exploratory and confirmatory studies in details. Similar standards of rigour are advisable for both forms of studies; this may be achieved by conforming to the general principles for the major domains that are outlined in Table 2 and incorporating these principles into a protocol that can be registered and published. It is important to note that both exploratory and confirmatory research can be closely linked: sometimes there can be exploratory and confirmatory components within the same studies. For example, a newly generated knockout mouse model is used to examine the effect of knockout on one specific phenotype (hypothesis testing–confirmatory) but may also describe a variety of other phenotypic characteristics as well (hypothesis generating–exploratory). Therefore, extra care in terms of applying these principles to reduce experimental bias would be needed before the commence of the studies. It also worth noting that sometimes it might not be compulsory or necessary to use some of the principles during exploratory studies such as sample size estimation and blinding which are albeit of highest importance in confirmatory research.

However, it is necessary to recognise how hypothesis confirming and hypothesis generating research relate to each other: while confirmatory research can turn into exploratory (e.g. if the findings are contrary to the hypothesis, this can lead to a new hypothesis that can be tested in a separate experiment), under no circumstances exploratory findings should be disseminated as the result of hypothesis confirming research by fitting a hypothesis to your results, i.e. to your *p*-values (often called HARKing = hypothesising after results are known or *p*-hacking = sifting through a multitude of *p*-values to find one below 0.05).

In conclusion, this chapter provides general concepts and principles that are important for the design and conduct of preclinical in vivo experiments, including experimental biases and how to reduce these biases in order to achieve the highest levels of rigour for hypothesis generating research using animals. The chapter should be used in conjunction with other relevant chapters in the handbook such as chapters “Blinding and Randomization”, “Minimum Information and Quality Standards for Conducting, Reporting, and Organizing In Vitro Research”, “Minimum Information in In Vivo Research”, “A Reckless Guide to *P*-Values: Local Evidence, Global Errors”, and “Quality Governance in Biomedical Research”.

## Figures and Tables

**Table 1:** Bias definition and bias-reducing methods (Lazic et al. 2018)

Name of bias	Definition of bias	Methods to reduce bias
Selection bias	Refers to the biased allocation of animals to different treatment groups, which could happen at the beginning of an animal study or at a stage where reassigning animals to different treatment groups is needed following an initial surgical procedure or treatment. Selection bias results in systematic differences in baseline characteristics between treatment groups (Higgins et al. 2011)	To avoid systematic differences between animals allocated to different treatment groups, one shall use a valid randomisation method, e.g. a randomisation software or even a simple method such as picking a number from a hat (Baastrup et al. 2010; Huang et al. 2013; Saghaei 2004). Detail for randomisation is covered in chapter “Blinding and Randomization”. Note that it is also necessary to conceal the allocation sequence from experimenters who will assign animals to treatment groups until the time of assignment
Performance bias	Related to the systematic differences in the care that is provided between different treatment groups or being exposed to factors other than the treatment that could influence the performance of the animals (Higgins et al. 2011; O’Connor and Sargeant 2014; van der Worp et al. 2010). Performance bias is a result of animals being managed differently due to, e.g. housing conditions, diet, group sizes per cage, location in the animal house, and experimenters who provide the care to animals are not blinded to treatment groups	One can avoid performance bias by improving the study design, e.g. applying the same housing, diet, location conditions to all the animals and by ensuring proper blinding of the experimenters to treatment groups, which keeps the experimenters who perform the experiment, collect data and access outcomes unaware of treatment allocation. Detail for blinding is covered in chapter “Blinding and Randomization”
Detection bias	Defined as the systematic distortion of the results of a study that occurs when the experimenter assessing behavioural outcome measures has the knowledge of treatment assignment to groups (van der Worp et al. 2010). In this circumstance, experimenters measuring the outcomes may introduce differential measurement of the outcomes rather than the treatment itself due to inadvertent expectation	The only way to avoid detection bias is a complete blinding of the experimenters, including those who analyse the data, so that they are not aware which animal(s) belong to which treatment group(s). The protocol should define at what stage the blinding codes will be broken (preferably only after data analysis has been completed). Detail for blinding is covered in chapter “Blinding and Randomization”
Attrition bias	Is the unequal occurrence and handling of deviations from protocol and loss to follow-up between treatment groups (van der Worp et al. 2010). This bias can occur when animals die or are removed from the study due to adverse effects of the treatment or pre-set criteria for removal before observing the outcomes; therefore, the outcomes are not observed for all animals, causing inadvertent bias (O’ Connor and Sargeant 2014)	Experimenters should report attrition information for each experimental group and also include outcomes that will not be affected by attrition. It is also advisable to consult a statistician to minimise the impact of attrition bias using some statistical approaches such as intention-to-treat analysis by imputing the missing data. Excluding “outliers” from analysis should be only undertaken as an extremely measure and should only be done to pre-stated criteria. Detail for statistics is covered in chapter “Blinding and Randomization”

**Table 2:** General descriptions for the major domains that contribute to experimental biases

Major domains	General descriptions
Sample size estimation	The sample size refers to the number of experimental units (e.g. a single animal, a cage of animals) per group. In hypothesis testing experiments, it should be determined with a power calculation. Studies that are not appropriately powered are unethical, and both underpowered and overpowered studies lead to a waste of animals. The former because they produce unreliable results and the latter because they use more animals than necessary
Randomisation	Refers to the steps to reduce systematic differences between comparison groups. Failure to conduct randomisation leads to selection bias
Allocation concealment	Refers to the practice of concealment of the group or treatment assignment (i.e. the allocation) and its sequence of each experimental unit from the experimenter until the time of assignment. Failure to conceal allocation will lead to selection bias. This should not be confused with randomisation
Blinding	Refers to the practice of preventing the experimenter who administer treatments, take care of the animals, assess the responses and analyse data from knowing the test condition. Failure of appropriate blinding leads to selection, performance and detection biases
Primary and secondary outcome measures	Primary outcome measure refers to the outcome measure of most interest, and it is related to the efficacy of an intervention that has the greatest importance for a given study. Secondary outcome measure refers to the outcome measure that is related to intervention efficacy but with less importance than the primary outcome measure and is used to evaluate additional intervention effects. It is important to declare what intervention effects are in the study protocol
Inclusion/exclusion criteria	Refers to criteria by which animals will be included or excluded in a given study, e.g. due to abnormal baselines or not reaching the required change in thresholds after designed experimental insult

**Table 3:** General principles to prevent experimental biases in hypothesis testing in vivo studies

Major domains	General principles
Sample size estimation	A power calculation (desired power of at least 0.8, and alpha = 0.05) to estimate the experimental group size should be carried out before any hypothesis testing study using pilot data or those relevant data from the literature. This could be done by using a statistical software. Detail on this can be found in chapter “A Reckless Guide to *P*-Values: Local Evidence, Global Errors”
Randomisation	There are different methods available to randomly allocate animals to experimental groups such as computer-generated randomisation. One should always consider to use the most robust, appropriate and available method for randomisation. Detail on this can be found in chapter “Blinding and Randomization”
Allocation concealment	Methods should be used to conceal the implementation of the random allocation sequence (e.g. numbered cages) until interventions are assigned, so that the sequence will not be known or predictable in advance by the experimenters involved in allocating animals to the treatment groups
Blinding	Blinding procedures should be carried out, so that the treatment identity should not be disclosed until after the outcome assessments have been finished for all animals and the primary analysis have been completed. In case that one experimenter conducts the whole study, any additional steps should be taken to preserve the blinding. Detail on this can be found in chapter “Blinding and Randomization”
Primary and secondary outcome measures	Experimenters should decide the outcome of great importance regarding the treatment efficacy before any study starts as the primary outcome measure. This is also usually used in the sample size estimation. Primary outcome measure cannot be changed once the study starts and when the results are known. Experimenters should also include secondary outcome measures relating to additional effects of treatments; these may be used for new hypothesis generating
Inclusion/exclusion criteria	Experimenters should set up the exact criteria which will include and exclude animals from their studies. Every animal should be accounted for, except under these criteria. They should be determined appropriately according to the study nature before the studies commence. Once determined, they cannot be changed during the course of investigation
